# ^13^C-tryptophan breath test detects increased catabolic turnover of tryptophan along the kynurenine pathway in patients with major depressive disorder

**DOI:** 10.1038/srep15994

**Published:** 2015-11-03

**Authors:** Toshiya Teraishi, Hiroaki Hori, Daimei Sasayama, Junko Matsuo, Shintaro Ogawa, Miho Ota, Kotaro Hattori, Masahiro Kajiwara, Teruhiko Higuchi, Hiroshi Kunugi

**Affiliations:** 1 Department of Mental Disorder Research, National Institute of Neuroscience, National Center of Neurology and Psychiatry, 4-1-1, Ogawahigashi, Kodaira, Tokyo, 187-8502, Japan; 2 Yokohama College of Pharmacy, 601 Matano-cho, Totsuka-ku, Yokohama, Kanagawa, 245-0066, Japan; 3 Tri-X Biomedical, Inc., 4-12-5-406, Minamiyawata, Ichikawa, Chiba, 272-0023, Japan; 4 National Center of Neurology and Psychiatry, 4-1-1, Ogawahigashi, Kodaira, Tokyo, 187-8551, Japan

## Abstract

Altered tryptophan–kynurenine (KYN) metabolism has been implicated in major depressive disorder (MDD). The l-[1-^13^C]tryptophan breath test (^13^C-TBT) is a noninvasive, stable-isotope tracer method in which exhaled ^13^CO_2_ is attributable to tryptophan catabolism via the KYN pathway. We included 18 patients with MDD (DSM-IV) and 24 age- and sex-matched controls. ^13^C-tryptophan (150 mg) was orally administered and the ^13^CO_2_/^12^CO_2_ ratio in the breath was monitored for 180 min. The cumulative recovery rate during the 180-min test (CRR_0–180_; %), area under the Δ^13^CO_2_-time curve (AUC; %*min), and the maximal Δ^13^CO_2_ (C_max_; %) were significantly higher in patients with MDD than in the controls (p = 0.004, p = 0.008, and p = 0.002, respectively). Plasma tryptophan concentrations correlated negatively with C_max_ in both the patients and controls (p = 0.020 and p = 0.034, respectively). Our results suggest that the ^13^C-TBT could be a novel biomarker for detecting a subgroup of MDD with increased tryptophan–KYN metabolism.

The kynurenine (KYN) pathway of l-tryptophan metabolism has gained increased attention in research into depression^1^,^2^,^3^,^4^,^5^,^6^,^7^. Indoleamine 2,3-dioxygenase (IDO) and tryptophan 2,3-dioxygenase (TDO, tryptophan pyrrolase) are the two initial and rate-limiting enzymes that convert tryptophan to KYN^1^,^3^,^8^,^9^. IDO is activated by proinflammatory cytokines, such as interferon (IFN)-α, IFN-γ, and tumor necrosis factor (TNF)-α^6^,^9^,^10^,^11^,^12^, which in addition to other inflammatory molecules such as interleukins, chemokines, and prostaglandins have been associated with major depressive disorder (MDD)^2^,^6^,^11^,^13^,^14^,^15^. The neuroactive KYN metabolites kynurenic acid (KA) and quinolinic acid (QUIN) are synthesized in the KYN–KA and KYN–nicotinamide adenine dinucleotide (NAD) pathways, respectively (Fig. 1). KA acts as an excitatory amino acid receptor antagonist, whereas QUIN has an excitotoxic effect. The activation of IDO promotes tryptophan catabolism in the KYN pathway and may cause reduced tryptophan availability for serotonin synthesis in the brain, resulting in imbalance or misregulation of the neuroactive metabolites. This mechanism may explain both MDD and the depressive symptoms observed in patients with inflammatory diseases and in those undergoing cytokine therapies^3^,^16^,^17^.

To clarify this mechanism, a number of clinical studies have used blood and cerebrospinal fluid (CSF) samples from patients undergoing cytokine therapy. These have evidenced decreased tryptophan levels, increased KYN levels, increased KYN/tryptophan ratios (used to estimate peripheral IDO activity), and correlations between depressive symptoms, and levels of tryptophan, and its metabolites^15^,^16^,^17^,^18^,^19^. However, contradictory results have also been reported^20^,^21^.

Several lines of evidence, including our recent meta-analysis^22^, have demonstrated decreased tryptophan levels in MDD^23^,^24^,^25^,^26^,^27^. However, it remains controversial whether the KYN pathway is activated in MDD. Indeed, although several studies have reported increased KYN/tryptophan ratios^23^,^28^,^29^, or its association with depression severity in MDD^30^, some negative results have also been reported^24^,^31^. These inconsistent findings, therefore, require further investigation. Furthermore, few studies have investigated the role of post-KYN metabolism: specifically, distinguishing between the KYN–KA and the KYN–NAD pathways in MDD^28^.

Cross-sectional quantitative data, obtained from peripheral blood or CSF samples, may provide a reasonable “snapshot”, reflecting total accumulation at the sampling time and site, but cannot measure changes in the synthesis or turnover rates of the metabolites. Indeed, these rates can alter without change in the total amount of metabolites in a dynamic steady-state equilibrium^32^. Additionally, metabolite turnover rates could be altered after any compensatory changes for the total amount of metabolites. Furthermore, because tryptophan, KYN and 3-hydroxykynurenine (3-OH-KYN) readily cross the blood–brain barrier (BBB)^33^,^34^,^35^, fluctuations in peripheral blood levels can directly affect metabolism of KYN pathway metabolites, including the synthesis of KA, 3-hydroxyanthranilic acid (3HAA), and QUIN in the brain, although these three metabolites do not readily cross the BBB^36^,^37^. IDO is distributed in various human tissues, including the intestine, lungs, placenta, kidneys, spleen, brain, and blood^8^, and when peripherally activated, IDO can affect the concentration of QUIN in the brain and CSF^38^. Raison *et al.* reported that there were significant positive correlations between peripheral and central measures of KYN and QUIN, but not tryptophan, and that peripheral tryptophan decreased while CSF tryptophan remained unchanged in patients receiving IFN-α therapy^16^. These factors point to the merit of monitoring KYN pathway activation in the whole body.

Tracer tests are advantageous for assessing *in vivo* metabolism of biological compounds as a more direct approach. Using a ^13^C-phenylalanine breath test, our group reported that altered phenylalanine metabolism could be identified in patients with schizophrenia^39^. Hankes *et al.* administered a ^14^C-tryptophan breath test to patients with scleroderma and reported that expiratory ^14^CO_2_ was decreased and labeled QUIN in the urine was increased^40^,^41^. Therefore, they suggested that metabolism after 3HAA may be altered in these patients. They also reported that the production of ^14^CO_2_ from ^14^C-labeled d-tryptophan was different from that of ^14^C-labeled l-tryptophan, and suggested that labeled l-tryptophan should be used for studies of tryptophan metabolism. In the present study, we used a stable isotope (i.e., non-radioactive tracer) in an l-[1-^13^C]tryptophan breath test (^13^C-TBT) to examine the whole-body kinetics and metabolism of orally administered tryptophan. In this method, the ^13^C label of l-[1-^13^C]tryptophan is released as ^13^CO_2_ when 3HAA, an immediate precursor for QUIN, is produced from tryptophan via the KYN–NAD pathway both peripherally and in the brain. This method also selectively observes the KYN–NAD pathway, but not the KYN–KA pathway, because ^13^CO_2_ is not produced during the synthesis of KA or xanthurenic acid. Therefore, ^13^CO_2_ formation measured by the ^13^C-TBT is an index of whole-body tryptophan metabolism via the KYN pathway and excludes the pathways for KA and xanthurenic acid production.

The aim of this study was to assess KYN pathway activity in patients with MDD. We hypothesized that the ^13^C-TBT would detect altered tryptophan metabolism via the KYN pathway in patients with MDD, and that the ^13^C-TBT indices would correlate negatively with plasma tryptophan concentrations.

## Methods

### Participants

We enrolled 18 patients with MDD (age range: 25–62 years; 10 women) and 24 healthy volunteers (age range: 21–61 years; 12 women), matched for age and sex, and who were biologically unrelated. Participants were recruited through advertisements in free local information magazines, by word of mouth, and via a website announcement; all were Japanese residents from the western part of Tokyo. Patients were also recruited from the National Center of Neurology and Psychiatry (NCNP) Hospital, Tokyo, Japan.

Consensus diagnoses were made for MDD by at least two experienced psychiatrists according to the criteria of the Diagnostic and Statistical Manual of Mental Disorders, Fourth Edition (DSM-IV)^42^, based on the Japanese version of the Mini-International Neuropsychiatric Interview (MINI)^43^, careful examination of medical records, observations, and additional unstructured interviews.

Healthy volunteers were screened by psychiatrists using unstructured interviews and the MINI. We confirmed that they had no history of psychiatric illness or contact with psychiatric services. Participants were excluded from both the patient and control groups if they met the DSM-IV criteria for intellectual disability, had a history of substance dependence or abuse, or had a history of severe head injury. We also excluded participants if they had a current serious physical disorder, particularly an inflammatory or liver disease (where tryptophan is primarily metabolized) or if they received any immunotherapy. All participants underwent blood tests for aspartate aminotransferase (AST), alanine aminotransferase (ALT), total protein, albumin, total bilirubin, blood urea nitrogen (BUN), and creatinine levels to exclude liver and kidney dysfunction. Individuals with abnormal blood test results were excluded.

The present study was conducted in accordance with the principles of the Declaration of Helsinki. The capacity of patients to understand the nature and purpose of the study and to provide consent was confirmed by an experienced psychiatrist. After explaining the study aim and protocol, signed informed consent was obtained from each participant. The study was approved by the ethics committee of the NCNP, Japan.

### Principle of the ^13^C-TBT

**Figure 1:**
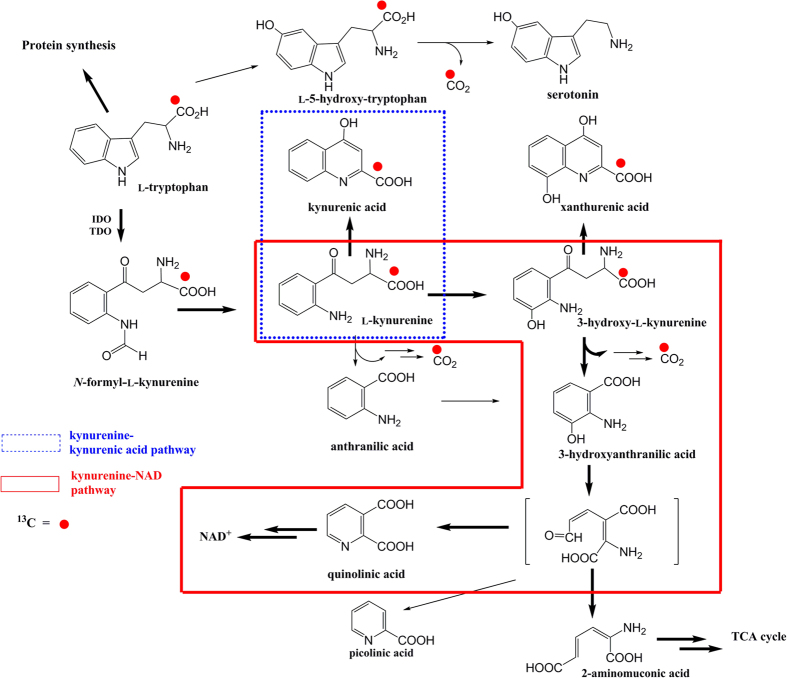
Schematic view of ^13^C-tryptophan metabolism. After utilization for protein synthesis, over 90% of dietary tryptophan is converted to KYN by one of two rate-limiting enzymes: by TDO in the liver under healthy conditions and by IDO in various human tissues (e.g., intestines, lungs, placenta, kidneys, spleen, blood, and brain) under inflammatory conditions. The ^13^CO2 derived from ^13^C-tryptophan is produced by the conversion of 3-OH-KYN into 3HAA via the KYN–NAD pathway (box with solid line) as the post-KYN metabolism. In addition, although ^13^CO2 is produced to a minor degree in the tryptophan–serotonin and anthranilic acid pathways, it is not produced by the KA (box with dotted line) or xanthurenic acid pathways. Bold arrows indicate quantitatively major pathways. Minor pathways and pathways without release of ^13^CO2 are omitted. Red circles indicate the ^13^C-labeled carbon. *Abbreviations:* IDO, indoleamine 2,3-dioxygenase; KA, kynurenic acid; KYN, kynurenine; NAD, nicotinamide adenine dinucleotide; TDO, tryptophan 2,3-dioxygenase; 3HAA, 3-hydroxyanthranilic acid; 3-OH-KYN, 3-hydroxy-l-kynurenine.

The kinetic values for l-[1-^13^C]tryptophan were assumed to be the same as for unlabeled tryptophan^44^. The following theory underpinned the ^13^C-TBT. After protein synthesis, dietary tryptophan, an essential amino acid for humans, is primarily metabolized along the KYN pathway in the liver, which accounts for over 90% of tryptophan catabolism^35^; in contrast, only 1%–3% of dietary tryptophan is metabolized via the serotonin pathway^34^. Under healthy conditions, the conversion of tryptophan to KYN via *N*-formyl-l-kynurenine is initiated by TDO, which is activated by stress and high glucocorticoid levels^1^,^2^,^45^. Thus, this is the product of elevated hypothalamic pituitary-adrenal (HPA) axis activity, which is often implicated in the development of depressive disorders^46^,^47^. In conditions where the immune system is stimulated, IDO is induced by proinflammatory cytokines^2^,^3^,^7^,^48^. Crucial to the test is that the ^13^CO_2_ derived from orally administered ^13^C-tryptophan can be produced by the conversion of 3-OH-KYN into 3HAA via the KYN–NAD pathway, but not by the KA or xanthurenic acid pathways (Fig. 1). The ^13^CO_2_ is also produced to much lesser degrees by minor pathways, such as the tryptophan–serotonin and anthranilic acid pathways. The ^13^CO_2_ measured by the ^13^C-TBT was, therefore, expected to reflect whole-body tryptophan–KYN–3HAA metabolism initiated by IDO or TDO.

The maximal Δ^13^CO_2_ (C_max_; %), time to reach the C_max_ (T_max_; min), area under the Δ^13^CO_2_-time curve (AUC; %*min), and the ratio of the total amount of exhaled ^13^CO_2_ to the administered dose of ^13^C-tryptophan (i.e., the cumulative recovery rate, CRR; %) were calculated from the Δ^13^CO_2_ (%) at each sampling point. Observations were made by infrared (IR) spectrometry in which IR absorption intensities of ^12^CO_2_ (2380 ± 10 cm^−1^) and ^13^CO_2_ (2280 ± 10 cm^−1^) were measured, as described previously^39^,^49^.

### Protocol for the ^13^C-TBT

The ^13^C-TBT was performed as previously described for the ^13^C-phenylalanine breath test^39^,^50^. Blood samples were collected after an overnight fast and stored at −20 °C until biochemical analysis (AST, ALT, total protein, albumin, total bilirubin, BUN, creatinine, and tryptophan levels) by the SRL Corporation (Tokyo, Japan). The plasma tryptophan level was measured by liquid chromatography–mass spectrometry. A dose of 150 mg/body of l-[1-^13^C]tryptophan (99 atom% ^13^C; Cambridge Isotope Laboratories, Cambridge, UK) was orally administered in 100 ml of water at 1000 h. Breath samples were collected in 250-ml breath-sampling bags (Otsuka Electronics Co., Ltd., Osaka, Japan) at 10, 15, 20, 30, 45, 60, 90, 120, 150, and 180 min after ingestion of ^13^C-tryptophan. The ^13^CO_2_ /^12^CO_2_ ratio in exhaled air was analyzed by IR spectrometry (UBiT-IR300 and UBiT-AS10, Otsuka Pharmaceutical, Tokyo, Japan).

### Clinical status and antidepressant medication

Current depression severity in patients was assessed using a 21-item version of the Hamilton Depression Rating Scale (HAMD-21) administered by a single experienced psychiatrist. Antidepressant doses were converted to imipramine-equivalent (IMIeq) doses according to published guidelines^51^,^52^.

### Statistical analysis

The Statistical Package for the Social Sciences (SPSS) version 11.0 (SPSS Japan, Tokyo, Japan) was used to conduct all statistical tests. Averages were expressed as mean ± standard deviation (SD). Statistical differences in the demographic data between groups were compared using the χ^2^ test and *t*-test for categorical variables and continuous variables, respectively. We then performed stepwise multiple regression analyses with two models, using the ^13^C-TBT indices (C_max_, AUC, and CRR_0–180_) as the dependent variable: in model 1, diagnosis, sex, age, and body weight were entered as possible predictors; and in model 2, diagnosis, sex, age, body weight, and plasma tryptophan concentration were entered as possible predictors.

A 2 × 10 (group × sampling point) repeated measures analysis of covariance (ANCOVA) was conducted to examine group differences in Δ^13^CO_2_ or CRR over time, controlling for sex, age, and body weight. Greenhouse–Geisser corrections were used for lack of sphericity. We used ANCOVA (controlling for sex, age, and body weight) and *t*-tests to compare ^13^C-TBT indices between the two groups. The T_max_, a discrete variable, was compared between the two groups using the Mann–Whitney *U* test. The correlations of each ^13^C-TBT index (AUC, CRR_0–180_, and C_max_) with the liver parameters and the plasma tryptophan concentrations were examined by Spearman’s and Pearson’s correlation analyses, respectively. Cramer’s *V*, Cohen’s *d*, η^2^, and partial η^2^ were calculated as measures of effect size for χ^2^ test, *t*-tests, ANOVAs, and ANCOVAs, respectively. In the patients, the correlations of each ^13^C-TBT index (AUC, CRR_0–180_, and C_max_) with IMIeq and HAMD-21 total score were examined using Spearman’s correlation analysis. A stepwise multiple regression was employed to assess independent predictors of C_max_, AUC, and CRR_0–180_, using sex, IMIeq, HAMD-21 total score, and plasma tryptophan concentration as candidate predictors. Statistical significance was two-tailed and set at p < 0.05.

## Results

### Demographic and clinical characteristics

The demographic and clinical characteristics of the patients and controls are presented in Table 1. There was no significant difference in sex, age, body weight, body mass index, education length, liver or kidney blood test results, or plasma tryptophan concentrations between the two groups.

### Predictors for ^13^C-TBT indices

Stepwise regression analyses were performed (Table 2). In model 1, diagnosis and sex were identified as significant predictors for C_max_, AUC, and CRR_0–180_. In model 2, diagnosis and sex remained significant predictors for C_max_, AUC, and CRR_0–180_, but the plasma tryptophan concentration was not a significant predictor in the model.

### Increased C_max_, AUC, and CRR in MDD

**Figure 2:**
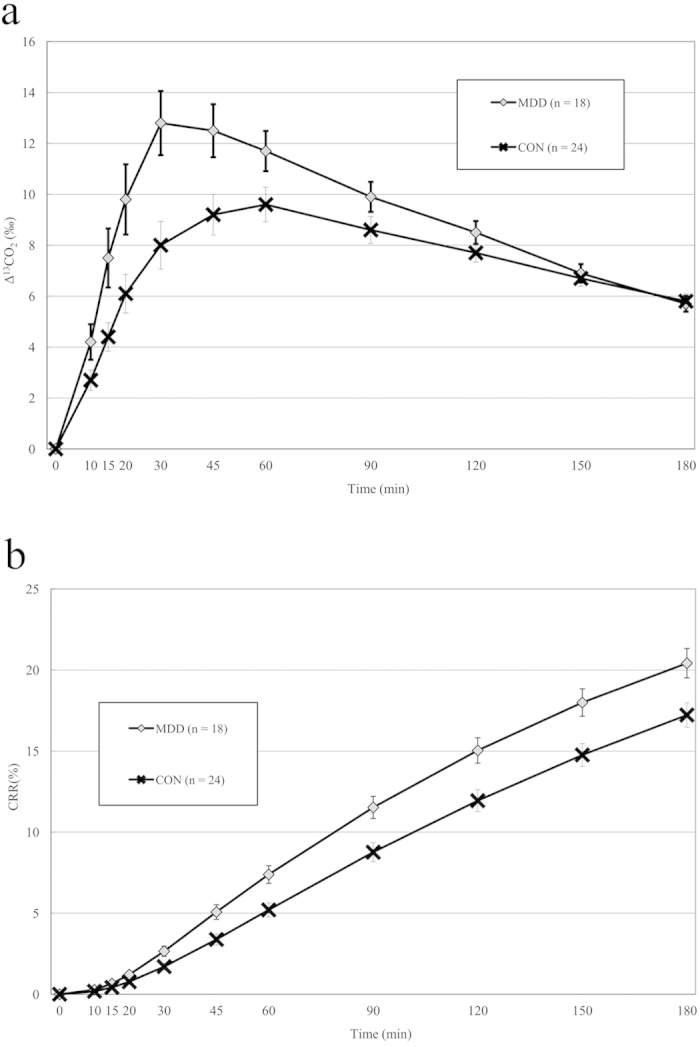
(**a**) Time course for the Δ^13^CO2 in expired air after the ^13^C-tryptophan breath test was applied to patients with major depressive disorder (MDD) and to healthy controls. (**b**) Time course for the cumulative recovery rate (CRR; %) in expired air for patients with MDD and for healthy controls. *Abbreviations:* MDD, major depressive disorder group; CON, control group; Time, time after ingesting the solution of ^13^C-tryptophan (150 mg).

Figure 2 shows the time course of mean values of Δ^13^CO_2_ (Fig. 2a) and CRR (Fig. 2b) in patients and controls. The repeated measures ANCOVA, controlling for sex, age, and body weight, indicated significant differences between the two groups in both Δ^13^CO_2_ and CRR over time. The repeated measures ANCOVA with Greenhouse–Geisser adjustment revealed significant interactions between the diagnosis and sampling point for both the Δ^13^CO_2_ and the CRR, indicating different patterns of change over time between the two groups. The Δ^13^CO_2_ and CRR values were higher in the patients than in the controls at all sampling points during the 180-min test (Fig. 2 and Table S1).

Using *t*-tests, the C_max_, AUC, and CRR_0–180_ were all significantly higher in the patients than in the controls, and these differences remained significant after ANCOVA controlling for sex, age, and body weight (Table 3). The T_max_ was reduced in the patients compared to the controls at the trend level.

### Association of ^13^C-TBT indices with tryptophan concentrations and clinical variables in patients

Both C_max_ (Pearson’s correlation coefficient [r] = −0.54, p = 0.020) and CRR_0–180_ (r = −0.48, p = 0.045) showed significantly negative correlations with plasma tryptophan concentrations in the patient group. In the controls, a significantly negative correlation was also observed for the plasma tryptophan concentrations with the C_max_ of the ^13^C-TBT (r = −0.43, p = 0.034). Further, stepwise multiple regression analysis demonstrated that plasma tryptophan concentrations were significant predictors of C_max_, while sex predicted the AUC and CRR_0–180_ (Table S2). In addition, both the AUC [Spearman’s rank correlation coefficient (ρ) = −0.53, p = 0.023] and CRR_0–180_ (ρ = −0.50, p = 0.033) were significantly and negatively correlated with the IMIeq dose. There were no significant correlations between ^13^C-TBT indices and depression severity (HAMD-21 score; data not shown). There were also no significant correlations between the ^13^C-TBT indices and the liver tests in the participants (Table S3).

## Discussion

We examined, for the first time, whole-body tryptophan metabolism using a ^13^C-TBT in patients with MDD and found that levels of exhaled ^13^CO_2_ were significantly increased in patients compared with healthy controls. Although plasma tryptophan concentrations were not significantly different between the two groups, tryptophan showed significant and negative correlations with the C_max_ in both the patient and control groups. In our ^13^C-TBT, exhaled ^13^CO_2_ is mainly attributable to the conversion of tryptophan via KYN into 3HAA. Therefore, our results support the study hypotheses and corroborate previous findings of KYN-pathway activation in MDD (i.e., the serotonin–KYN hypothesis)^48^,^53^.

The ^13^CO_2_ measured by our ^13^C-TBT can be produced by either the KYN pathway or the serotonin pathway (Fig. 1). Although just 1%–3% of dietary tryptophan is metabolized via the serotonin pathway^34^, expiration of ^14^CO_2_ (produced by the conversion of ^14^C-labeled 5-hydroxytryptophan to serotonin) can be measurably decreased in depression^54^. Therefore, in our patients with MDD, the observed increase in expired ^13^CO_2_ could be attributed to increased metabolism in the KYN pathway rather than the serotonin pathway. Specifically, the observed increase in ^13^CO_2_ could relate to increased conversion of 3-OH-KYN to 3HAA (Fig. 1), suggesting an increased production of KYN and 3-OH-KYN (precursors of 3HAA), as well as QUIN (the immediate catabolite of 3HAA). Importantly, 3-OH-KYN and 3HAA have neurotoxic effects that are mediated by oxidative stress^2^,^55^. In addition, QUIN not only has neurotoxic effects mediated by the activation of NMDA-type glutamate receptors^2^,^56^ but also induces lipid peroxidation and oxidative stress^57^. Therefore, our results are consistent with the neurodegeneration hypothesis of MDD^48^. Although tryptophan, KYN, and 3-OH-KYN are able to cross the BBB^33^,^34^,^35^, KA, 3HAA, and QUIN cannot do so readily^36^,^37^. It has also been reported that there are significant positive correlations between peripheral and central measures of the KYN and QUIN, but not tryptophan^16^. Further studies are warranted to elucidate the relationship between concentrations of peripheral and central KYN pathway metabolites and indices of ^13^C-TBT. These neurotoxic metabolites in the KYN pathway are also implicated in several neurodegenerative disorders^11^,^12^,^58^. It may, therefore, be interesting to examine the possible clinical use of the ^13^C-TBT in these disorders.

We observed significant increases in ^13^C-TBT indices in the patient group, but found no significant difference in the plasma tryptophan concentrations between patients and controls. This result is inconsistent with previous data, including the results of our own meta-analysis^22^,^23^,^24^,^25^,^26^,^27^. The inconsistency may have occurred due to the small sample size and the modest severity of depression in our patients (mean HAMD-21 score of 12.6), as well as the modest effect size. Indeed, our meta-regression analysis found a negative correlation between plasma tryptophan concentrations and depression severity^22^. Moreover, some previous studies have reported no significant difference in plasma tryptophan levels between patients with MDD and healthy controls^59^,^60^,^61^. Our results may suggest that ^13^C-TBT is more sensitive than plasma tryptophan levels at detecting increased metabolism in the KYN pathway.

As shown in Table 3, differences existed between the 2 groups in most of the pharmacokinetic parameters, including the AUC, CRR_0–180_, and C_max_, but not the T_max_. All these parameters are affected by gastric emptying, digestive absorption, metabolism rates, and respiratory status^62^. The AUC reflects the global amount of exhaled ^13^CO_2_ during the test session. However, although the CRR_0-180_ also reflects the global process, it may be a better index because it takes into account the body surface area. The CRR may therefore be the best parameter for biomarker research in depression, particularly given the high statistical significance. Finally, while T_max_ is particularly subject to the influence of digestion, C_max_ could be more relevant because it more closely reflects the metabolic rate.

There are several limitations to this study. First, the sample size was small and the patients with MDD had only modestly severe disease. In addition, depression severity was not measured for the control sample. Despite conflicting results^31^, there is normal variability in mild depression symptoms in the healthy population together with evidence of a relationship between depressive symptoms and the KYN/tryptophan ratio^30^. Such methodological limitation might have minimized, rather than exaggerated the difference between the MDD and control groups in ^13^C-TBT indices. Further studies are needed to address these issues, and should include larger samples, patients with more severe forms of MDD, and assessment for depressive symptoms among healthy controls. A second limitation is that we included 6 patients who had co-morbid diagnoses of dysthymia or an anxiety disorder. There is some evidence suggesting that there is a different KYN metabolism pathway between anxiety and depression^63^. To examine the possible influence of the comorbidity on the current results, we re-analyzed the data, excluding the 6 patients with MDD comorbidities. However, the results were essentially unchanged and the significant differences in ^13^C-TBT indices remained between the 2 groups (data not shown). In addition, some studies have shown positive associations of suicidality with KYN and QUIN^29^,^64^,^65^,^66^. Almost all of the patients in our study (17 of 18) had no history of having attempted suicide. However, because the MDD group in our study might be the heterogeneous group with different biological mechanisms, further studies will be needed with a sample of MDD patients without comorbidity or suicidal behavior. Third, only 10 patients were drug-free, with a further 8 being antidepressant-treated. The AUC and CRR_0–180_ correlated negatively with the IMIeq dose. Therefore, because some antidepressants have anti-inflammatory effects^67^, inhibit TDO^68^, or can affect KYN/tryptophan^31^, it is possible that antidepressant treatment minimized the differences in the indices of the ^13^C-TBT and concentrations of plasma tryptophan between the patients and controls. Fourth, although we measured plasma tryptophan, we did not measure any KYN pathway metabolites. Unfortunately, no conclusive relationship can be determined between the measures in this study and the ^13^C-TBT indices, without knowing the data for other KYN pathway metabolites. In addition, we did not obtain data on inflammatory markers such as C-reactive protein, proinflammatory cytokines, or cortisol levels, which may affect depressive symptoms through the KYN pathway^31^. Thus, additional studies are required to examine the relationship between these data and the ^13^C-TBT indices. Lastly, we did not assess the effects of vitamin B6 deficiency, which can interfere with the breakdown of KYN^69^. Pyridoxal phosphate, the active form of vitamin B6, is needed for the conversions of KYN to anthranilic acid and KA, and for that of 3-OH-KYN to 3HAA and xanthurenic acid.

In conclusion, the ^13^C-TBT, a noninvasive tracer test, provided evidence of increased tryptophan metabolism along the KYN–NAD pathway in patients with MDD. Consistent with this, ^13^C-TBT indices correlated negatively with plasma tryptophan concentrations. Therefore, whole-body tryptophan metabolism detected by our ^13^C-TBT could be a novel and sensitive biomarker for the diagnosis and subtyping of MDD, with the potential to enhance the development of new drugs for the treatment of MDD. Moreover, when ^13^C-TBT is combined with the ^13^C-phenylalanine breath test^39^, discrimination between MDD and schizophrenia may be possible.

## Additional Information

**How to cite this article**: Teraishi, T. *et al.*^13^C-tryptophan breath test detects increased catabolic turnover of tryptophan along the kynurenine pathway in patients with major depressive disorder. *Sci. Rep.* **5**, 15994; doi: 10.1038/srep15994 (2015).

## Supplementary Material

Supplementary Information

## Figures and Tables

**Table 1:** Demographic and clinical characteristics of patients with major depressive disorder, and healthy controls.

	Patients (n = 18)	Controls (n = 24)	statistic analysis
Sex, male: n (%)	8 (44.4)	12 (50.0)	χ^2^(1) = 0.13, p = 0.76, Cramer’s *V* = 0.06
Age (year)	39.9 ± 10.1	45.1 ± 11.9	t(40) = 1.49, p = 0.14, Cohen’s *d* = 0.46
Body weight (kg)	60.3 ± 9.4	63.0 ± 15.9	t(38.1) = 0.68, p = 0.50, Cohen’s *d* = 0.20
Body mass index (kg/m^2^)	22.5 ± 2.6	22.8 ± 4.3	t(38.5) = 0.34, p = 0.73, Cohen’s *d* = 0.10
Education (year)	15.1 ± 2.2	14.2 ± 2.1	t(40) = 1.41, p = 0.17, Cohen’s *d* = 0.44
Comorbid dysthymia/anxiety disorders (n)	6		
HAMD-21 total score	12.6 ± 7.6		
IMIeq (mg per day)	69.4 ± 114.9		
Tryptophan (μmol l^−1^)	49.6 ± 7.4	49.7 ± 10.1	t(40) = 0.05, p = 0.96, Cohen’s *d* = 0.02
AST (U l^−1^)	19.4 ± 6.5	19.0 ± 5.2	t(40) = 0.22, p = 0.83, Cohen’s *d* = 0.07
ALT (U l^−1^)	12.9 ± 7.6	14.4 ± 8.1	t(40) = 0.62, p = 0.54, Cohen’s *d* = 0.19
Total protein (g dl^−1^)	7.3 ± 0.3	7.4 ± 0.4	t(40) = 0.51, p = 0.61, Cohen’s *d* = 0.16
Albumin (g dl^−1^)	4.7 ± 0.3	4.5 ± 0.3	t(40) = 1.74, p = 0.09, Cohen’s *d* = 0.54
Total bilirubin (mg dl^−1^)	0.6 ± 0.2	0.5 ± 0.2	t(40) = 0.74, p = 0.47, Cohen’s *d* = 0.23
BUN (mg dl^−1^)	13.7 ± 2.7	12.2 ± 3.3	t(40) = 1.59, p = 0.12, Cohen’s *d* = 0.49
Cr (mg dl^−1^)	0.73 ± 0.16	0.71 ± 0.15	t(40) = 0.38, p = 0.71, Cohen’s *d* = 0.12

*Abbreviations:* HAMD-21, 21-item version of the Hamilton Depression Rating Scalecreatinine; IMIeq, Imipramine-equivalent antidepressant dose; BUN, blood urea nitrogen; Cr, creatinine.

**Table 2:** Stepwise multiple regression for l-[1-^13^C]tryptophan breath test indices as dependent variables.

Dependant variable	Significant predictor variable^a^	Adjusted R^2^	Standardized Coefficient (β)	p value
Model 1				
C_max_	sex^b^	0.36	0.436	0.001
	diagnostic status^c^		0.426	0.002
AUC	sex^b^	0.48	0.617	<0.001
	diagnostic status^c^		0.316	0.008
CRR_0–180_	diagnostic status	0.25	0.378	0.008
	sex		0.361	0.011
Model 2				
C_max_	sex^b^	0.36	0.436	0.001
	diagnostic status^c^		0.426	0.002
AUC	sex^b^	0.48	0.617	<0.001
	diagnostic status^c^		0.316	0.008
CRR_0–180_	diagnostic status	0.25	0.378	0.008
	sex		0.361	0.011

*Abbreviations:* C_max_, the maximal Δ^13^CO_2_ (%); AUC, area under the Δ^13^CO_2_-time curve (min %); CRR_0-180_, the cumulative recovery rate during the 180 min test (%).

^a^Possible predictor variables. Model 1: diagnostic status, sex, age, and body weight; model 2: diagnostic status, sex, age, body weight, and plasma tryptophan concentration.

^b^Sex was measured on a nominal scale: 1 = male; 2 = female.

^c^Diagnostic status was measured on a nominal scale: 1 = healthy control; 2 = major depressive disorder.

**Table 3:** l-[1-^13^C]tryptophan breath test results in patients with major depressive disorder and healthy controls.

Parameter	Patients (n = 18) Mean ± SD	Controls (n = 24) Mean ± SD	*t–*test/Mann–Whitney *U* test	ANCOVA^a^
C_max_	14.5 ± 4.3	10.9 ± 3.1	p = 0.003 Cohen’s *d* = 0.99	p = 0.002 partial η^2^ = 0.23
AUC	1596.2 ± 405.3	1320.0 ± 350.2	p = 0.023 Cohen’s *d* = 0.74	p = 0.008 partial η^2^ = 0.18
CRR_0-180_	20.4 ± 3.8	17.2 ± 3.7	p = 0.009 Cohen’s *d* = 0.86	p = 0.004 partial η^2^ = 0.21
T_max_^b^	30	45	p = 0.068^c^ r = 0.28	

*Abbreviations:* C_max_, the maximal Δ^13^CO_2_ (%); T_max_, time to reach the C_max_ (min); AUC, area under the Δ^13^CO_2_-time curve (min %); CRR_0-180_, the cumulative recovery rate during the 180 min test (%); r, the effect size of the Mann–Whitney *U* test.

^a^Healthy controls versus patients with major depressive disorder in analysis of covariance controlled for sex, age, and body weight.

^b^Median.

^c^Healthy controls versus patients with major depressive disorder by the Mann–Whitney *U* test.
