# Supplementary material for: 13C-tryptophan breath test detects increased catabolic turnover of tryptophan along the kynurenine pathway in patients with major depressive disorder
Source: Sci Rep. 2015 Nov 3;5:15994. doi: 10.1038/srep15994 (PMC4630584; doi:10.1038/srep15994)
Supplement: Supplementary Information [file srep15994-s1.doc]

**^13^C-tryptophan breath test detects increased catabolic turnover of tryptophan along the kynurenine pathway in patients with major depressive disorder**

Running title: ^13^C-tryptophan breath test in major depressive disorder

Toshiya Teraishi^1^, Hiroaki Hori^1^, Daimei Sasayama^1^, Junko Matsuo^1^, Shintaro Ogawa^1^, Miho Ota^1^, Kotaro Hattori^1^, Masahiro Kajiwara^2, 3^, Teruhiko Higuchi^4^, Hiroshi Kunugi^1^,*

^1^ Department of Mental Disorder Research, National Institute of Neuroscience, National Center of Neurology and Psychiatry, 4-1-1, Ogawahigashi, Kodaira, Tokyo, 187-8502, Japan

^2^ Yokohama College of Pharmacy, 601 Matano-cho, Totsuka-ku, Yokohama, Kanagawa, 245-0066, Japan

^3^ Tri-X Biomedical, Inc., 4-12-5-406, Minamiyawata, Ichikawa, Chiba, 272-0023, Japan

^4^ National Center of Neurology and Psychiatry, 4-1-1, Ogawahigashi, Kodaira, Tokyo, 187-8551, Japan

**Table S1. Repeated measures analysis of covariance in patients with major depressive disorder and healthy controls: change in Δ^13^CO_2_ and CRR for 180 min of L-[1-^13^C]tryptophan breath test**

| parameter |  | Patients (n = 18) | | |  | Controls (n = 24) | | |  | Repeated measures ANCOVA ^b^ | |
| --- | --- | --- | --- | --- | --- | --- | --- | --- | --- | --- | --- |
|  |  | Mean ± SD | | |  | Mean ± SD | | |  | F | P |
|  |  |  |  |  |  |  |  |  |  |  |  |
| Δ^13^CO_2_ |  |  |  |  |  |  |  |  |  |  |  |
| Δ^13^CO_2_ at 10 min (‰) |  | 4.2 | ± | 3.0 |  | 2.7 | ± | 2.0 |  |  |  |
| Δ^13^CO_2_ at 15 min (‰) |  | 7.5 | ± | 4.9 |  | 4.4 | ± | 2.8 |  |  |  |
| Δ^13^CO_2_ at 20 min (‰) |  | 9.8 | ± | 5.9 |  | 6.1 | ± | 3.7 |  |  |  |
| Δ^13^CO_2_ at 30 min (‰) |  | 12.8 | ± | 5.3 |  | 8.0 | ± | 4.6 |  |  |  |
| Δ^13^CO_2_ at 45 min (‰) |  | 12.5 | ± | 4.4 |  | 9.2 | ± | 3.9 |  |  |  |
| Δ^13^CO_2_ at 60 min (‰) |  | 11.7 | ± | 3.3 |  | 9.6 | ± | 3.3 |  |  |  |
| Δ^13^CO_2_ at 90 min (‰) |  | 9.9 | ± | 2.5 |  | 8.6 | ± | 2.6 |  |  |  |
| Δ^13^CO_2_ at 120 min (‰) |  | 8.5 | ± | 1.9 |  | 7.7 | ± | 1.8 |  |  |  |
| Δ^13^CO_2_ at 150 min (‰) |  | 6.9 | ± | 1.5 |  | 6.7 | ± | 1.5 |  |  |  |
| Δ^13^CO_2_ at 180 min (‰) |  | 5.7 | ± | 1.3 |  | 5.8 | ± | 1.4 |  |  |  |
| Interaction ^a^ |  |  |  |  |  |  |  |  |  | 4.56 | 0.011 |
| Between group |  |  |  |  |  |  |  |  |  | 9.32 | 0.004 |
|  |  |  |  |  |  |  |  |  |  |  |  |
| CRR |  |  |  |  |  |  |  |  |  |  |  |
| CRR at 10 min (%) |  | 0.3 | ± | 0.2 |  | 0.2 | ± | 0.1 |  |  |  |
| CRR at 15 min (%) |  | 0.7 | ± | 0.5 |  | 0.4 | ± | 0.3 |  |  |  |
| CRR at 20 min (%) |  | 1.2 | ± | 0.8 |  | 0.8 | ± | 0.5 |  |  |  |
| CRR at 30 min (%) |  | 2.7 | ± | 1.3 |  | 1.7 | ± | 1.0 |  |  |  |
| CRR at 45 min (%) |  | 5.1 | ± | 1.9 |  | 3.4 | ± | 1.7 |  |  |  |
| CRR at 60 min (%) |  | 7.4 | ± | 2.3 |  | 5.2 | ± | 2.2 |  |  |  |
| CRR at 90 min (%) |  | 11.5 | ± | 2.9 |  | 8.8 | ± | 2.9 |  |  |  |
| CRR at 120 min (%) |  | 15.0 | ± | 3.3 |  | 11.9 | ± | 3.3 |  |  |  |
| CRR at 150 min (%) |  | 18.0 | ± | 3.6 |  | 14.8 | ± | 3.5 |  |  |  |
| CRR at 180 min (%) |  | 20.4 | ± | 3.8 |  | 17.2 | ± | 3.7 |  |  |  |
| Interaction ^a^ |  |  |  |  |  |  |  |  |  | 8.82 | 0.003 |
| Between group |  |  |  |  |  |  |  |  |  | 11.66 | 0.002 |
|  |  |  |  |  |  |  |  |  |  |  |  |

CRR, the cumulative recovery rate.

^a^ Interaction between diagnostic status and sampling point.

^b^ Repeated measures analysis of covariance with sex, age and body weight as covariates.

**Table S2. Stepwise multiple regression for L-[1-^13^C]tryptophan breath test indices as dependent variables in patients with major depressive disorder**

|  | | | | | | |  |  |  |
| --- | --- | --- | --- | --- | --- | --- | --- | --- | --- |
| Dependant variable |  | predictor variable ^a^ |  | Adjusted R^2^ |  | Standardized Coefficient (β) |  | p value |  |
|  |  |  |  |  |  |  |  |  |  |
| C_max_ |  | tryptophan |  | 0.25 |  | −0.54 |  | 0.020 |  |
|  |  | sex |  |  |  | 0.34 |  | 0.145 |  |
|  |  | HAMD-21 |  |  |  | −0.37 |  | 0.082 |  |
|  |  | IMIeq |  |  |  | −0.13 |  | 0.603 |  |
|  |  |  |  |  |  |  |  |  |  |
| AUC |  | sex |  | 0.39 |  | 0.65 |  | 0.004 |  |
|  |  | tryptophan |  |  |  | −0.16 |  | 0.456 |  |
|  |  | HAMD-21 |  |  |  | −0.17 |  | 0.382 |  |
|  |  | IMIeq |  |  |  | −0.31 |  | 0.111 |  |
|  |  |  |  |  |  |  |  |  |  |
| CRR_0-180_ |  | sex |  | 0.26 |  | 0.55 |  | 0.017 |  |
|  |  | tryptophan |  |  |  | −0.30 |  | 0.193 |  |
|  |  | HAMD-21 |  |  |  | 0.03 |  | 0.902 |  |
|  |  | IMIeq |  |  |  | −0.32 |  | 0.126 |  |
|  |  |  |  |  |  |  |  |  |  |
|  |  |  | | | | |  |  |  |

C_max_, the maximal Δ^13^CO_2_ (‰); AUC, area under the Δ^13^CO_2_-time curve (min･‰); CRR_0-180_, the cumulative recovery rate during the 180 min test (%).

^a^ Possible predictor variables included sex, plasma tryptophan concentration (tryptophan), total score of 21-item version of the Hamilton Depression Rating Scale (HAMD-21), and imipramine-equivalent antidepressant dose (IMIeq). Sex was measured on a nominal scale: 1 = male; 2 = female.

Significant predictor variables are underlined.

**Table S3. Correlations between each L-[1-^13^C]tryptophan breath test index and each liver blood test value**

|  |  |  | CRR_0-180_ | |  | AUC | |  | C_max_ | |  |
| --- | --- | --- | --- | --- | --- | --- | --- | --- | --- | --- | --- |
|  |  | N | ρ | P |  | ρ | P |  | ρ | P |  |
| *Major depressive disorder* | |  |  |  |  |  |  |  |  |  |  |
|  | AST (U l^−1^) | 18 | −0.08 | 0.77 |  | −0.05 | 0.84 |  | 0.21 | 0.41 |  |
|  | ALT (U l^−1^) |  | −0.14 | 0.58 |  | −0.18 | 0.48 |  | 0.14 | 0.58 |  |
|  | Total protein (g dl^−1^) |  | −0.28 | 0.26 |  | −0.32 | 0.20 |  | −0.19 | 0.44 |  |
|  | Albumin (g dl^−1^) |  | −0.36 | 0.14 |  | −0.45 | 0.06 |  | −0.41 | 0.09 |  |
|  | Total bilirubin (mg dl^−1^) |  | −0.32 | 0.20 |  | −0.17 | 0.51 |  | −0.26 | 0.30 |  |
| *Control* | |  |  |  |  |  |  |  |  |  |  |
|  | AST (U l^−1^) | 24 | 0.15 | 0.47 |  | 0.04 | 0.85 |  | 0.13 | 0.54 |  |
|  | ALT (U l^−1^) |  | 0.36 | 0.09 |  | 0.04 | 0.84 |  | 0.16 | 0.47 |  |
|  | Total protein (g dl^−1^) |  | 0.18 | 0.41 |  | 0.22 | 0.30 |  | 0.23 | 0.27 |  |
|  | Albumin (g dl^−1^) |  | 0.08 | 0.69 |  | −0.01 | 0.97 |  | 0.07 | 0.73 |  |
|  | Total bilirubin (mg dl^−1^) |  | −0.19 | 0.37 |  | −0.34 | 0.11 |  | −0.27 | 0.20 |  |
| *Total* | |  |  |  |  |  |  |  |  |  |  |
|  | AST (U l^−1^) | 42 | 0.02 | 0.89 |  | −0.01 | 0.97 |  | 0.16 | 0.31 |  |
|  | ALT (U l^−1^) |  | 0.09 | 0.56 |  | −0.06 | 0.70 |  | 0.10 | 0.52 |  |
|  | Total protein (g dl^−1^) |  | −0.04 | 0.80 |  | 0.01 | 0.96 |  | 0.05 | 0.76 |  |
|  | Albumin (g dl^−1^) |  | −0.01 | 0.97 |  | −0.08 | 0.62 |  | 0.02 | 0.91 |  |
|  | Total bilirubin (mg dl^−1^) |  | −0.16 | 0.30 |  | −0.20 | 0.20 |  | −0.17 | 0.28 |  |

AUC, area under the Δ^13^CO_2_-time curve (min･‰); CRR_0-180_, the cumulative recovery rate during the 180 min test (%); C_max_, the maximal Δ^13^CO_2_ (‰); N, number of participants; ρ, Spearman's correlation coefficient.
